# Uterine Lipoleiomyoma: A Case Report and Review of the Literature

**DOI:** 10.7759/cureus.54112

**Published:** 2024-02-13

**Authors:** Wedd K Alharthi, Amal Khalil, Sultana Alnejaimi, Bayan Hafiz

**Affiliations:** 1 Department of Anatomic Pathology, King Faisal Specialist Hospital and Research Centre, Jeddah, SAU; 2 Department of Anatomic Pathology, King Fahad Armed Forces Hospital, Jeddah, SAU; 3 Department of Anatomic Pathology, King Abdulaziz Medical City, Jeddah, SAU; 4 Department of Anatomic Pathology, Ministry of Health, Maternity and Children Hospital, Makkah, SAU

**Keywords:** smooth muscle, uterine fatty tumors, postmenopause, leiomyoma variant, uterine lipoleiomyoma

## Abstract

Uterine leiomyomas are the most common benign neoplasms found in women of reproductive age. Lipoleiomyoma, a rare variant of leiomyomas, is composed of intermixed smooth muscle cells and mature adipocytes. These neoplasms are usually discovered incidentally in obese, perimenopausal, or postmenopausal women. In this report, we present a case of lipoleiomyoma in a postmenopausal woman who presented with vaginal bleeding and back pain.

## Introduction

Lipoleiomyoma is a rare variant of uterine leiomyoma, with an incidence ranging from 0.03% to 0.2% [1]. First discovered by Lopstein in 1916 [2], this condition is morphologically characterized by the presence of smooth muscle bundles admixed with mature adipocytes [3]. Various studies have classified this tumor both as a uterine fatty tumor, subdivided into “lipoma” and “mixed lipoma/leiomyoma” (lipoleiomyoma), or as a manifestation of metaplastic changes in leiomyoma [4]. Despite these varying theories, lipoleiomyomas generally follow a benign clinical course [5].

Typically, the symptoms of lipoleiomyoma are similar to those of conventional leiomyomas, which can range from being asymptomatic to causing pelvic pain or vaginal bleeding [5]. While the common site of lipoleiomyoma is the uterine corpus, rare cases have also been reported in the cervix, ovary, broad ligament, and retroperitoneum [6].

In this report, we detail a case of a postmenopausal female presenting with vaginal bleeding. Both gross and microscopic examinations revealed features indicative of lipoleiomyoma.

## Case presentation

A 62-year-old postmenopausal female presented to the obstetrics and gynecology clinic with complaints of vaginal bleeding and a yearlong history of back pain. She experienced menopause at 55 years of age. On examination, the patient was conscious, oriented, alert, and vitally stable. The chest and abdominal examinations were unremarkable. Initial laboratory tests were all within the normal range. Gynecological examination of the cervix, vulva, and vagina showed no clinical abnormalities. Abdominal and pelvic computerized tomography (CT) scans revealed multiple well-circumscribed uterine intramural masses, with the largest measuring 2 cm × 1 cm × 1.2 cm. The patient subsequently underwent a total abdominal hysterectomy and a bilateral salpingo-oophorectomy.

Post-operatively, in the gross examination, the uterus and cervix measured 8 cm × 5 cm × 4.4 cm and contained multiple well-circumscribed intramural masses ranging from 2.3 cm to 0.5 cm in maximum dimension. The cut surfaces of these masses showed a homogenous, whorled appearance with focal yellowish areas resembling a fat component, the largest of which measured 1.6 cm in maximum dimension. There were no signs of gross necrosis or hemorrhage. The right fallopian tube measured 5 cm × 1.5 cm, the left fallopian tube measured 4.8 cm × 1.5 cm, and both tubes are unremarkable. The right ovary measured 3 cm × 2 cm, and the left ovary was 4 cm × 1.8 cm. Both ovaries are grossly normal.

**Figure 1:**
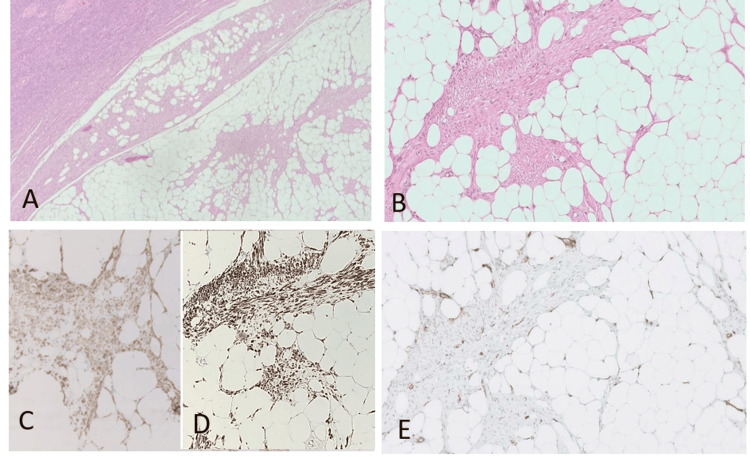
Histopathology examination with hematoxylin and eosin (H&E) stains and Immunohistochemistry (A and B) A well-demarcated fatty tumor with intersecting smooth muscle bundles with bland-looking nuclei and no necrosis or mitosis present (H&E, 40× and 100×); (C and D) SMA and Desmin both are strong positive (IHC, 100×); (E) CD34 is negative (IHC, 100×).

Microscopic evaluation of the intramural masses revealed bland, spindle-shaped smooth muscle cells arranged in intersecting fascicles, admixed with mature fat cells. There was no evidence of nuclear atypia or necrosis and only minimal mitotic activity (1 per 10 high-power fields) (Figure 1A-1B). Immunohistochemistry studies were performed using markers including smooth muscle actin (SMA), desmin, and CD34. The smooth muscle cells were diffusely positive for SMA and desmin (Figure 1C-1D), while CD34 was negative (Figure 1E). These findings are consistent with the diagnosis of a benign lipoleiomyoma. Two weeks post-surgery, the patient was doing well without further symptoms or complaints.

## Discussion

Uterine lipoleiomyoma is a very rare benign neoplasm typically diagnosed during histopathological examination. Malignant transformation is extremely rare, and only one case of leiomyoliposarcoma has been reported [7]. Lipoleiomyoma occurs predominantly in postmenopausal women aged 50 to 80 years [8]. Although most patients are asymptomatic, some present with symptoms similar to those of conventional leiomyomas, such as vaginal bleeding, abdominal discomfort, pelvic and lower back pain, frequent urination, and constipation [5]. Imaging plays an important role in determining the nature of this fat-containing lesion [1]. MRI with a fat-suppression sequence is particularly effective in diagnosing uterine lipoleiomyoma due to its high sensitivity and specificity for fat and multisectional ability to locate the lesion precisely [1]. However, it is not always possible to differentiate uterine lipoleiomyoma from other uterine lipomatous tumors [1]. Macroscopically, the tumor appears as a well-circumscribed, solid gray-tan mass with yellow discoloration, indicating the presence of adipose tissue elements.

The pathogenesis of lipoleiomyoma remains controversial, with theories suggesting it is formed during leiomyoma differentiation, the lipomatous metamorphosis of a preexisting leiomyoma, or newly formed in the uterine myometrium [9,10]. Estrogen deficiency may also play a role in inducing and promoting the proliferation of the lipomatous component in lipoleiomyoma [11]. Typically, lipoleiomyomas that are asymptomatic and clinically similar to leiomyomas do not require specific treatment [4]. The primary surgical treatment of these tumors is hysterectomy [4], as in our case. Depending on the patient’s symptoms and fertility goals, other modalities, such as uterine artery embolization and myomectomy, can be performed [12]. Generally, lipoleiomyomas follow a benign clinical course. However, there have been rare reports of lipoleiomyosarcomas arising from uterine lipoleiomyomas [13], which can be distinguished from their malignant counterparts by the bland appearance of the nuclei and occasional mitosis in the smooth muscle component [14]. Lipoleiomyoma has been reported with other uterine pathologies such as adenomyosis, endometriosis, endometrial hyperplasia, and polyps [14].

## Conclusions

Lipoleiomyoma is a rare type of leiomyoma characterized by the presence of adipocytes. These tumors follow a benign course and do not affect mortality. The final diagnosis of lipoleiomyoma is established based on a histopathological test. The primary treatment is surgical, typically with no documented risk of recurrence.
